# Interaction in Spoken Word Recognition Models: Feedback Helps

**DOI:** 10.3389/fpsyg.2018.00369

**Published:** 2018-04-03

**Authors:** James S. Magnuson, Daniel Mirman, Sahil Luthra, Ted Strauss, Harlan D. Harris

**Affiliations:** ^1^Department of Psychological Sciences, University of Connecticut, Storrs, CT, United States; ^2^Connecticut Institute for the Brain and Cognitive Sciences, University of Connecticut, Storrs, CT, United States; ^3^Department of Psychology, University of Alabama at Birmingham, Birmingham, AL, United States; ^4^McConnell Brain Imaging Centre, McGill University, Montreal, QC, Canada

**Keywords:** psycholinguistics, spoken word recognition, computational models, speech perception, Bayesian models, simulations

## Abstract

Human perception, cognition, and action requires fast integration of bottom-up signals with top-down knowledge and context. A key theoretical perspective in cognitive science is the *interactive activation hypothesis:* forward and backward flow in bidirectionally connected neural networks allows humans and other biological systems to approximate optimal integration of bottom-up and top-down information under real-world constraints. An alternative view is that online feedback is neither necessary nor helpful; purely feed forward alternatives can be constructed for any feedback system, and online feedback could not improve processing and would preclude veridical perception. In the domain of spoken word recognition, the latter view was apparently supported by simulations using the interactive activation model, TRACE, with and without feedback: as many words were recognized more quickly *without* feedback as were recognized faster with feedback, However, these simulations used only a small set of words and did not address a primary motivation for interaction: making a model robust in noise. We conducted simulations using hundreds of words, and found that the majority were recognized more quickly with feedback than without. More importantly, as we added noise to inputs, accuracy and recognition times were better with feedback than without. We follow these simulations with a critical review of recent arguments that online feedback in interactive activation models like TRACE is distinct from other potentially helpful forms of feedback. We conclude that in addition to providing the benefits demonstrated in our simulations, online feedback provides a plausible means of implementing putatively distinct forms of feedback, supporting the interactive activation hypothesis.

## Introduction

A central question in cognitive science is how sensory data should be integrated with prior knowledge. Bi-directional (i.e., bottom-up and top-down) information flow is one solution. Such “interactive activation" allows early and continuous access to prior knowledge, which can tune perceptual systems to prior and conditional probabilities based on experience (MacDonald et al., 1994; Knill and Richards, 1996; McClelland et al., 2006). In addition to providing a basis for effects of top-down knowledge, feedback provides an efficient means for resolving ambiguous, noisy, or obscured inputs (McClelland and Rumelhart, 1981). McClelland et al. (2014) describe this *interactive activation hypothesis* very clearly:

*Interactive activation hypothesis.* Implementation of perceptual and other cognitive processes within bidirectionally connected neural networks in the brain provides the mechanism that addresses the key computational challenges facing perceptual systems, and it gives rise to the approximate conformity of human performance to optimal perceptual inference in real time (p. 6).

**FIGURE 1:**
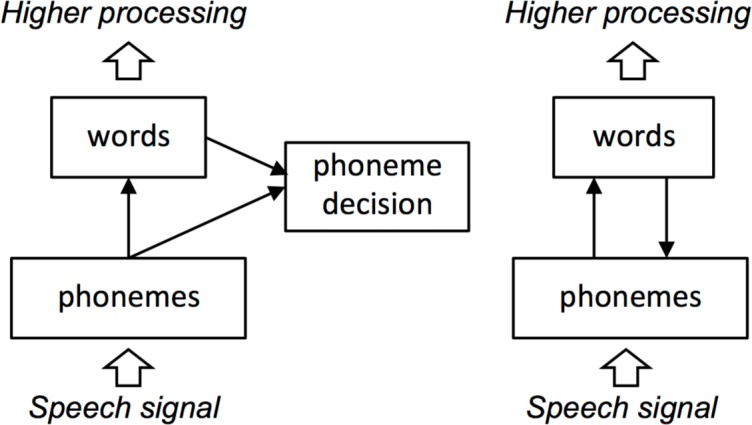
Schematics of autonomous (Left) and interactive (Right) models of spoken word recognition. In autonomous models, lexical effects result from post-perceptual integration of perceptual and lexical substrates. In interactive models, lexical effects on phonological-level behavior result from direct influence of lexical knowledge on perceptual substrates. The large arrows delimit the scope of most current models, which are concerned primarily with mapping an abstract representation of the speech signal (phonemes, or in the case of TRACE, distributed features over time) to word forms.

An alternative view holds that feedback cannot improve performance, feedback *only* accounts for effects of top-down knowledge, and bottom-up and top-down information can be more effectively integrated without feedback (e.g., Norris et al., 2000; see **Figure 1** for schematics of interactive and autonomous models). In the specific case of spoken word recognition, proponents of such “autonomous” or “modular” views have asserted that feedback is not necessary and, furthermore, could never help word recognition – and might in fact hinder it. As Norris et al. (2000) put it:

The best performance that could possibly be expected from a word recognition system is to reliably identify the word whose lexical representation best matches the input representation. This may sound trivially obvious, but it highlights the fact that a recognition system that simply matched the perceptual input against each lexical entry, and then selected the entry with the best fit, would provide an optimal means of performing isolated word recognition (independent of any higher-level contextual constraints), limited only by the accuracy of the representations. Adding activation feedback from lexical nodes to the input nodes (whether phonemic or featural) could not possibly improve recognition accuracy at the lexical level (p. 301).

Norris et al. (2016) reiterate their arguments (Norris et al., 2000) against feedback in interactive activation models (IAMs), but also make a strong case that there are other kinds of feedback that could benefit processing. They use the term “activation feedback” to refer to feedback in IAMs and argue that it differs qualitatively from helpful forms of feedback, such as feedback for learning, feedback for attentional control, of feedback in service of generative models such as predictive coding. In our “General Discussion,” we will critically review their taxonomy of feedback types and the claim that activation feedback stands alone as a theoretically unhelpful form of feedback. First, though, it is crucial to put the claim that feedback cannot benefit recognition to the test.

Norris et al. (2000) bolstered the argument that activation feedback cannot benefit performance by citing a set of simulations reported by Frauenfelder and Peeters (1998; FP98 from here on) using the interactive-activation TRACE model (McClelland and Elman, 1986). In these simulations, feedback modified phonemic processing, but it did not have a “general beneficial effect on word recognition.” FP98 compared the time it took a target word in TRACE to reach an activation threshold with feedback set to the default level or turned off (set to 0.0; their Simulation 5). They compared recognition times (RTs) for a set of 21 words that had been chosen for other simulations. The expectation was that feedback would speed recognition, since the bottom-up input should be amplified by recurrence via top-down connections. However, FP98 found that about half their items were recognized more quickly without feedback than with feedback. The implication would seem to be that feedback in TRACE is a mechanism added simply to account for top-down effects, rather than a mechanism serving a useful purpose (benefiting recognition) from which top-down effects emerge. Further, if the interactive architecture in TRACE serves no helpful function, this would raise doubts about the plausibility of a helpful, let alone necessary, role of feedback.

However, the FP98 simulation does not address the crucial issue of whether feedback makes a model robust under degraded conditions, such as the presence of external or internal noise (McClelland and Rumelhart, 1981; McClelland and Elman, 1986). Primary characteristics of parallel distributed processing systems include *graceful degradation* under cases of noisy, distorted, obscured, or otherwise incomplete inputs (McClelland et al., 1986). Noise is a crucial ecological consideration, given considerable internal noise in neural systems and the variable (and often literally noisy) conditions under which speech is experienced (see extended discussion in McClelland and Elman, 1986). Here, we test the possibility that the apparent failure of activation feedback to benefit processing in TRACE stems from the failure to test the model in conditions where bottom-up information does not perfectly identify a lexical target – that is, under conditions of noise. We will also consider the possibility that the result may not generalize beyond the 21-word subset of the TRACE lexicon FP98 tested. A benefit of feedback in TRACE simulations, especially given increasing levels of noise, would support interactivity as a viable candidate mechanism for approximating optimal inference under uncertainty. However, such a finding by itself would not suffice to address claims made by Norris et al. (2016) that activation feedback in IAMs is qualitatively different from other forms of feedback; we address this issue in the section “General Discussion.”

## Simulations: Feedback and Noise

We reexamined the role of feedback in TRACE (and, by extension, other interactive models) by comparing word recognition in TRACE with and without feedback, and under levels of increasing noise. This allowed us to test the prediction that feedback in interactive-activation models should make them robust to noise (that is, feedback should allow the model to maintain higher accuracy and speed as noise is added to inputs). We also tested the generality of the FP98 failure to find a feedback advantage without noise by testing every word in the standard, 211-word TRACE lexicon and every word in a new, larger, 907-word lexicon (details described below). FP98 only tested 21 words with homogenous characteristics (seven-phoneme words with uniqueness point at phoneme position four), which had been selected for other simulations. It is possible that these words were not representative of the entire lexicon with respect to the effects of feedback.

Although more than 30 years have passed since the introduction of TRACE, it remains the model of speech perception and spoken word recognition that accounts for the broadest and deepest set of empirical phenomena (for reviews, see Magnuson et al., 2012; Mirman, 2016). In addition to phenomena that TRACE captured at its outset, such as categorical perception and top-down lexical effects (McClelland and Elman, 1986), TRACE accounts for various phenomena that were documented after TRACE first appeared, including fine-grained time course indexed by eye tracking (Allopenna et al., 1998; Dahan et al., 2001a,b) and mouse tracking (Spivey et al., 2005), individual differences due to acquired (Mirman et al., 2011) and developmental language disorders (McMurray et al., 2010), and aspects of typical language development (Mayor and Plunkett, 2014). As a result, TRACE continues to be a central cognitive model of spoken word recognition. As such, it offers the ideal testbed for empirical evaluation of interactive activation as a mechanism for robust word recognition under uncertainty.

### Methods

#### Lexicons

FP98 developed a significantly larger lexicon than the original TRACE lexicon in order to begin to test whether TRACE would be robust as the lexicon size increases. We did not have access to FP98’s expanded lexicon of 1,024 words, so we generated our own based primarily on the procedures FP98 describe for compiling a larger lexicon: we scanned a large electronic dictionary (20,000 words) for all items that could be transcribed using only TRACE’s 14 phonemes (/p/, /b/, /t/, /d/, /k/, /g/, /s/, /∫/, /r/, /l/, /a/, /i/, /u/, /ˆ/). This yielded approximately 500 words. Substituting /ˆ/ for schwa and /a/ for /æ/, and collapsing across vocalic and consonantal liquids (substituting /l/ for vocalic /L/ and /r/ for vocalic /R/) brought the total to 907. In the rest of this paper, we will refer to this lexicon as *biglex*. For the sake of more direct comparability with previous work, we also conducted all simulations with the original 211-word TRACE lexicon, *slex*. This allowed us to examine whether any patterns of results are an artifact of scaling up the lexicon.

Readers may be concerned that biases of some sort may have influenced the selection of words for the lexicons. We do not think this is plausible. Consider the criteria McClelland and Elman (1986) report for selecting the original 211 words.

TRACE … has detectors for the 211 words found in a computerized phonetic word list that met all of the following constraints: (a) the word consisted only of the phonemes listed above; (b) it was not an inflection of some other word that could be made by adding “-ed,” “-s,” or “-ing”; (c) the word together with its “-ed,” “-s,” and “-ing” inflections occurred with a frequency of 20 or more per million in the Kučera and Francis (1967, p. 19) word count.

These criteria are quite generic, and there is no discernible bias linking these selection criteria with those we described for *biglex.* Both lexicons provide sufficiently large sampling from English that we do not believe that any details of the results that follow could be artifacts of item selection (although artifacts could arise were we to use significantly smaller lexicons).

#### TRACE Parameters

We used the standard (McClelland and Elman, 1986) settings for *slex*, but modified two parameters for *biglex*. FP98 reported that changing the lexical inhibition parameter from the standard 0.030 to 0.025 and feedback from 0.030 to 0.015 improved performance with a large lexicon; we used the FP98 adjusted parameters for *biglex*. There are several reasons that we did not “re-explore” the TRACE parameter space for our simulations. First, McClelland and Elman (1986, p. 22) reported that changes to TRACE’s default parameters changed the size and timing of some effects, but broad ranges of values generated patterns of behavior similar to those that they report in their series of simulations (p. 22), so there is no reason to suspect that our findings depend critically upon the default parameters. Second, TRACE has simulated many additional findings since the original 1986 report (Strauss et al., 2007; Magnuson et al., 2012, 2013, for reviews) using the default *slex* lexicon and default parameters; deviations from the default parameters with *slex* would require confirming that TRACE could still account for all prior findings with the new parameter settings. Finally, while FP98 adjusted the parameters for a larger lexicon based on extensive explorations conducted by Peeters (1989, Unpublished), we have also conducted our *biglex* simulations with the default *slex* parameters and the results do not differ qualitatively.

#### Noise

Gaussian noise was sampled from a normal distribution function and added independently to each element of the input stimulus vector for each time step (cf. McClelland, 1991). We created seven levels of noise with mean of 0.0 and standard deviation ranging from 0.0 to 1.5 in steps of 0.25. Our aim was to have noise values that would impede the model very slightly at one end of the continuum and greatly at the other; as we shall see shortly, this range of noise provided this.

#### Operationalizing Recognition

**FIGURE 2:**
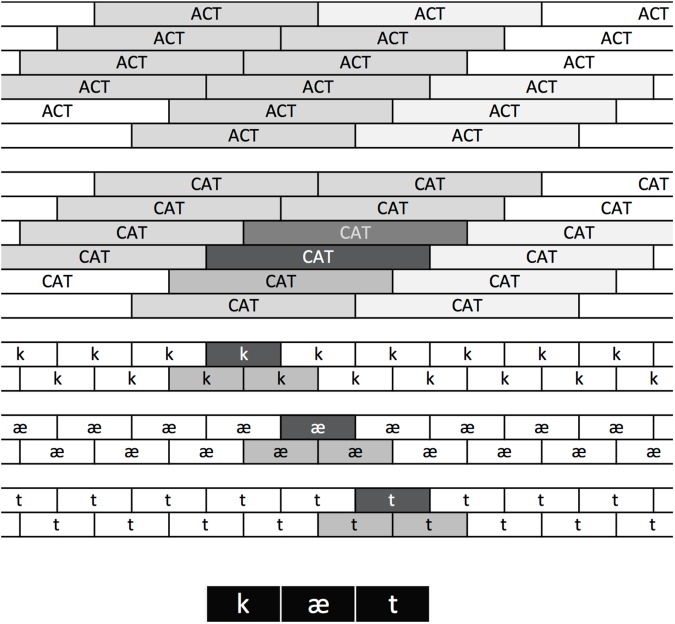
The TRACE reduplication architecture. At the bottom, featural input is replaced with squares indicating where the feature patterns corresponding to the ordered phonemes of CAT occur in a sample utterance. Feature patterns activate phonemes aligned with them in the phoneme layer, where there are copies of each phoneme are tiled over time. The copy maximally aligned with the /k/ features is strongly activated (indicated by its dark background). Adjacent copies overlap partially and are partially activated. The same principles apply at the word layer, where the copy of CAT maximally aligned with the strongly aligned /k/, /æ/, and /t/ units is strongly activated, with graded activation of other CAT and ACT units depending on their degree of overlap. Note that because /t/ and /k/ share many features, word units that overlap at offset with CAT still become moderately activated.

TRACE solves the phoneme and word segmentation problem (how to parse a sequence into discrete phonemes at one level and words at the next) by having copies of each phoneme and word unit aligned at regular intervals over time (**Figure 2**). For example, if the model is set up to process 33 time steps worth of input, then there are 33 copies of the templates corresponding to each word. There is one copy aligned with each of the possible onsets of the word in the input (though note this is the length of the memory “trace” constructed for the model; the elements in that trace – e.g., the word units aligned at slice 4 – can continue processing beyond 33 time steps). Given this reduplication, a modeler must decide how to interpret the bank of word units. FP98 began by basing their interpretation on the method McClelland and Elman (1986) used; one simply chooses the units known to be aligned with the input. FP98 point out that the unit immediately to the “right” of the perfectly aligned unit (that is, the unit aligned with the following time step) sometimes attains a high activation, and therefore they summed the activation of the target unit perfectly aligned with the input and the unit immediately following it. One must also decide how to treat potential competitors. FP98 considered any units overlapping in time with the target units to be competitors. FP98 then calculated response probabilities at each TRACE processing step using the Luce (1959) choice rule:

(1) $$R_{i}=\frac{e^{{\mathrm{ka}}_{i}}}{\sum \;\;e^{{\mathrm{ka}}_{j}}\;}$$

where *R_i_* is the response probability for item *i*, *a_i_* is that item’s activation in TRACE, *k* is a constant (set to 20 in the FP98 simulations) that controls target-competitor separation, and the summed activations in the denominator include all target and competitor units. In the FP98 simulations, an item was considered “recognized” when its response probability exceeded a threshold of 0.9.

We deviated from the FP98 procedure in a few ways. First, we minimized selection criteria, allowing any word node to be included; we did not preselect maximally aligned words nor limit the set of potential competitors. One reason to preselect targets is because of the large number of computations required to compute response strength (*R*) for all copies of all words at every time step (for example, with 33 copies of 211 words over 100 time steps, the simulation of each word would require computing *R* for 211 × 211 × 33 × 100 = 146,919,300 values). An examination of activation values revealed that typically only approximately 20–30 word units reached activations greater than 0.05, and word units in this set were aligned with the first few time slices. We conservatively reduced the set of units to include in the choice rule to the top 50 units (rather than 20–30) from the first 10 time slices in any simulation. Thus, for a given simulation, we only computed *R* values for the 50 word units with the highest peak activations (and computed *R* for those units over 100 time steps). Note that this reduction is simply a convenience for data analysis; all copies of all words were of course operational during the actual simulations.

We then conducted simulations with *slex* and *biglex* to find the combination of *k* and response probability accuracy threshold that would maximize accuracy without noise and without feedback. We found that the ideal combination (which maximized hits and minimized false alarms) for both *slex* and *biglex* was *k* set to 13 and response probability threshold set to 0.5. Without noise and without feedback, these parameters yielded proportions correct of 0.93 for *slex* and 0.92 for *biglex* (alternative operational definitions for correct responses might allow higher accuracy, but we chose this definition for the sake of simplicity). Finding these maximizing values is a simple matter of calculating proportion correct with a range of parameter combinations (where simulations are scored as correct only when the target and no other word exceeds the threshold). We examined a range of 2–20 for *k* and from 0.01 to 0.99 for the response probability threshold.

#### Simulation and Analysis Software

Initial simulations used jTRACE, a Java reimplementation of the original TRACE C code^1^ (Strauss et al., 2007). For purposes of speed (particularly with the larger lexicon), final versions of simulations used the original C code with minor modifications. Simulation results were processed using a series of Perl preprocessing scripts and R analysis and plotting scripts. Revised C code, Perl and R scripts are available from JSM, and will soon be available from http://magnuson.psy.uconn.edu.

#### Procedure

Simulations were run in batches where every word in *slex* or *biglex* was used once as the target. For each lexicon, we ran 14 batches of simulations: each of the seven levels of noise crossed with feedback (set to the lexicon-appropriate parameter) or no feedback. In each case, the decision rule described above was applied and RT and accuracy were recorded. Simulations were run for 100 cycles.^2^

### Predictions

#### Recognition for Clear Inputs

FP98 reported that feedback appeared not to facilitate word recognition in TRACE, since equal numbers of their 21 words were recognized more quickly without feedback as were recognized more quickly with feedback. If feedback serves no useful purpose, we should replicate this result when we examine recognition of all words in *slex* (the original 211-word TRACE lexicon) and *biglex* (our 907-word lexicon). If instead feedback is generally helpful in TRACE, we should find that, on average, words are recognized more quickly with feedback than without.

#### Recognition in Noise

Feedback allows integration of current input and prior knowledge, not just in the explicit sense of top-down effects but also in the implicit sense of optimal inference under uncertainty. That is, feedback provides a generative model (cf. McClelland, 2013) meant to promote robust processing in cases of missing or noisy inputs. Therefore, we make two predictions with respect to noise-added inputs in TRACE. (1) Even if a general feedback advantage on RT is not observed in noise-free inputs, we expect one to emerge as noise levels increase. (2) We expect accuracy to be better preserved with feedback than without.

### Results

**FIGURE 3:**
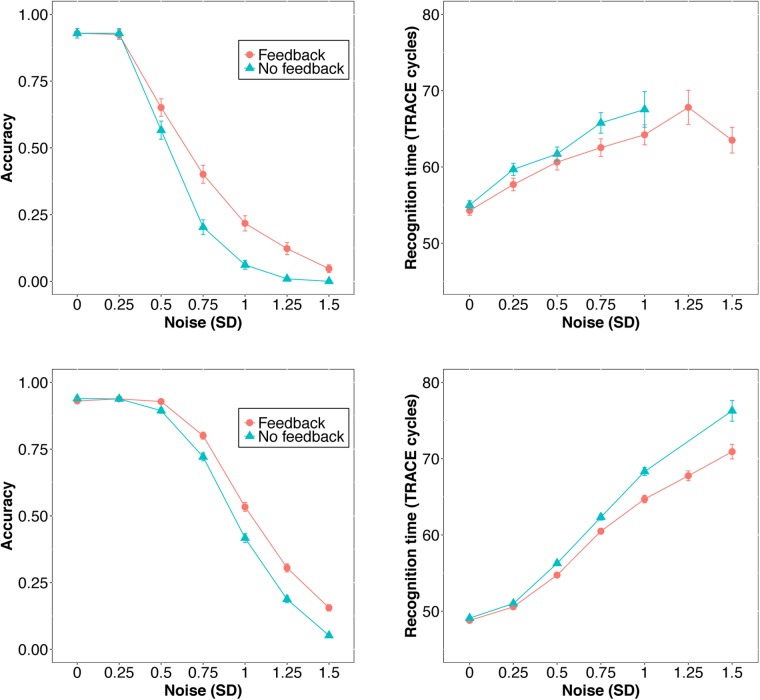
Accuracy (left) and RT (right) as a function of feedback level and noise in the original 211-word TRACE lexicon, *slex* (top) and the new 907-word lexicon, *biglex* (bottom). Error bars span ±1 standard error (and are too small to be seen for some datapoints).

**FIGURE 4:**
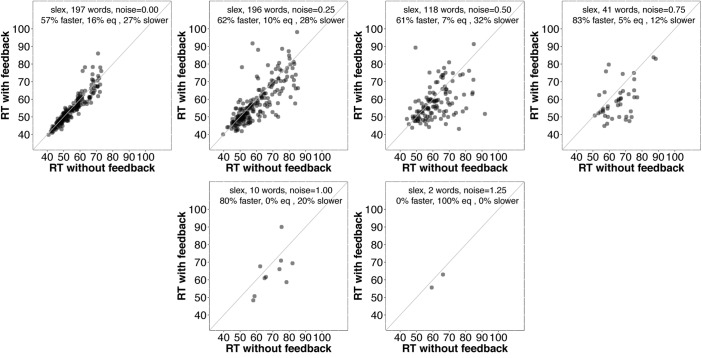
Scatterplots of RTs for words recognized correctly with and without feedback in *slex* with no noise (top left) and progressively greater noise. The final panel (bottom right) is missing because no words were recognized without feedback at the highest noise level (*SD* = 1.5). Points below the identity line are words that were recognized more quickly with feedback than without; points above the identity line are words that were recognized more slowly with feedback than without. Annotations indicate the percentages of words recognized more quickly with feedback, more slowly with feedback, or equivalently quickly with and without feedback. At every noise level, the proportion of words recognized more quickly with feedback than without is greater than the proportion recognized more slowly with feedback.

First, we examined average accuracy and RT with and without feedback as noise increased for *slex* (top row, **Figure 3**) and *biglex* (bottom row, **Figure 3**). For both lexicons, there were clear average benefits of feedback: Feedback promoted greater accuracy and faster RTs as noise was added. Next, we examined the impact of feedback at the level of individual items. Scatterplots for items in *slex* that were correctly recognized with feedback on and feedback off at progressive levels of noise are shown in **Figure 4**. Items recognized more quickly with feedback are plotted below the identity line. Without noise (top left panel), 27% of words were recognized more quickly without feedback, while the rest were recognized more quickly with feedback (57%), or had equivalent RTs with and without feedback (16%). Thus, without noise, feedback tends to speed up word recognition, although a minority of items are recognized more quickly without feedback.

**FIGURE 5:**
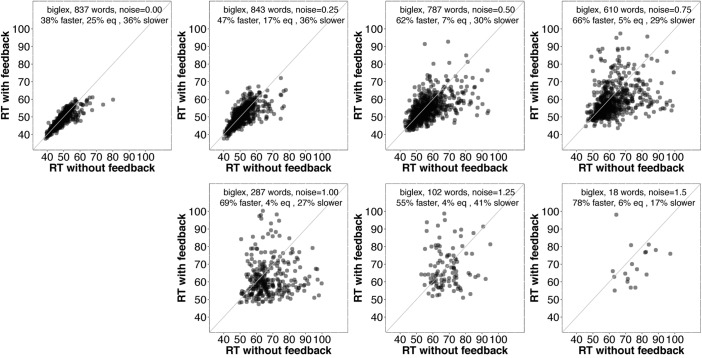
Scatterplots of RTs for words recognized correctly with and without feedback in *biglex* with no noise (top left) and progressively greater noise. The proportion of words recognized more quickly with feedback is greater than the proportion recognized more slowly with feedback at every noise level.

As noise increases, the number of items included decreases since we are only plotting items recognized correctly with and without feedback. There is a clear trend for even more items to be recognized more quickly with feedback than without as noise increases. **Figure 5** contains analogous plots for *biglex*. The same trends are present: As noise increases, so does the proportion of words recognized more quickly with feedback than without.

**FIGURE 6:**
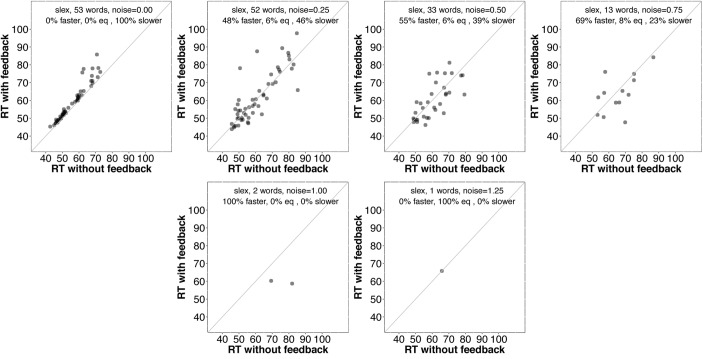
Scatterplots of RTs for words recognized correctly with and without feedback in *slex*, limited to words that were recognized *more slowly* with feedback than without when no noise was added (top left). The final panel (bottom right) is missing because no words were recognized without feedback at the highest noise level (*SD* = 1.5). On average, then, even words that exhibit a feedback *disadvantage* without noise show a benefit from feedback as noise increases.

**FIGURE 7:**
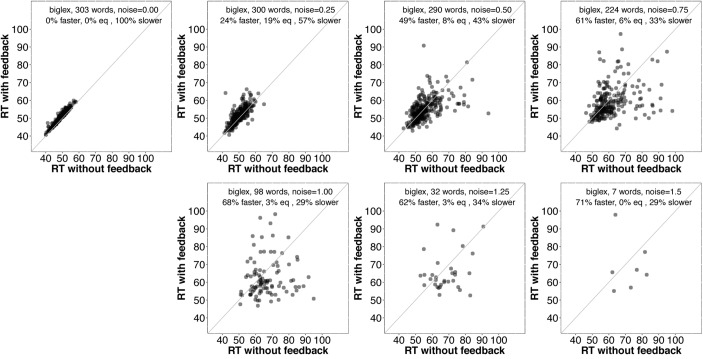
Scatterplots of RTs for words recognized correctly with and without feedback in *biglex*, limited to words that were recognized *more slowly* with feedback than without when no noise was added (top left). As with *slex* (**Figure 5**), on average, even words that exhibit a feedback *disadvantage* without noise show a benefit from feedback as noise increases.

It is unclear why some words are recognized more quickly without feedback. Before considering why this might be the case, it would be interesting to know whether those items tend to benefit from feedback as noise is added. **Figures 6** and **7** provide scatterplots relevant for this question for *slex* and *biglex*, respectively. In both figures, the top left panel is restricted to items that were recognized more quickly without feedback in the absence of noise (hence, all points are above the identity line). Subsequent panels are restricted to the “survivors” from these subsets (that is, the items that were still recognized both with and without feedback) as noise increased. Again, there is a clear trend.^3^ As noise is added, feedback promotes faster recognition, even for items that were recognized more slowly with feedback in the absence of noise.

### Discussion

In summary, feedback facilitates word recognition. On average, feedback preserves accuracy and boosts recognition speed for words presented in noise (**Figure 3**). At the level of specific items, in the absence of noise, only a minority are recognized more quickly without feedback than with feedback (most are recognized more quickly or equally quickly with feedback). As noise increases, a greater proportion of words are recognized more quickly with than without feedback – even for words that were recognized more quickly *without* feedback in the absence of noise.

But why are *any* items recognized more slowly with feedback than without? Again, in the absence of noise, recognition is slower with feedback for 27% of words in *slex* and 36% of words in *biglex*. In broad strokes, the effects of feedback on a word depend on (1) recurrent lexical-phoneme-lexical boosts from lexical feedback and (2) inhibition from other activated words. The feedback boost can speed recognition, while inhibition can slow it. The fact that feedback usually helps recognition implies that the boost is *usually* more potent than increased competition resulting from feedback.

In cases where there is no advantage with or without feedback (16 and 25% with *slex* and *biglex*, respectively), the feedback boost and feedback inhibition are in balance. For a feedback disadvantage to occur the feedback boost must be overwhelmed by increased competition – i.e., the boost must help competitors more than the target, allowing them to send sufficient inhibition to the target to slow its recognition compared to the no-feedback condition. Strauss (2006) used extensive TRACE simulations to analyze the conditions under which this occurs.

One way for a feedback disadvantage to occur is if a short word is embedded in the target; for example, “cow” is embedded in “couch.” When the model is presented with the input “couch,” it must encounter the pattern corresponding to “cow” first. TRACE exhibits a short-word advantage because longer words have more sites for inhibition (words are only inhibited by words that temporally overlap with or are adjacent to them; Magnuson et al., 2013, for a review and detailed explanation of activation and competition in TRACE). However, we cannot appeal to embedding as a general principle to explain feedback disadvantages: Many words with embeddings still exhibit a feedback advantage. Rather, Strauss (2006) found that a conspiracy of several factors appears to be required. Feedback disadvantages tend to be observed when two or more of the following hold: onset embeddings, negative ganging, particular combinations of cohort size and target length, and particular combinations of constituent phonemes. We will focus on negative ganging here, as Strauss (2006)’s simulations indicate that negative ganging is a key determinant of feedback disadvantages and suggest that cohort size, target length and phonetic saliency can modulate the size of negative ganging effects but are themselves unlikely to drive feedback disadvantages.

**FIGURE 8:**
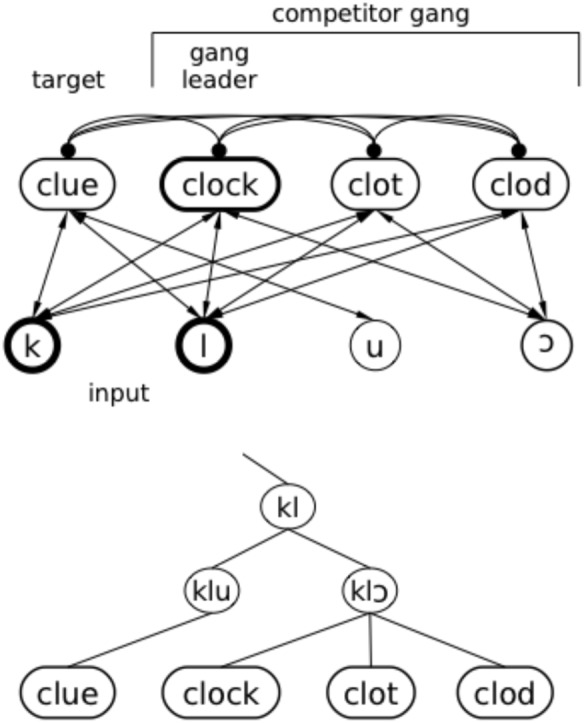
Illustration of how feedback can occasionally hurt. As the system “hears” *clue*, the initial /kl/ activates the “*clock* gang;” each word initially overlapping with *clock* contributes to mutual reinforcement of the gang, and each gang member temporarily inhibits *clue*. In the top panel, excitatory connections (arrows) and inhibitory connections (filled circles) are illustrated with a standard network diagram. In the lower panel, an implicit tree structure based on partial overlap is illustrated. Although all the items are represented in TRACE at the same level (as in the top panel), the tree structure illustrates the overlap and embedding relationships that lead to gang effects.

Negative ganging occurs when a target (e.g., *clue*) overlaps partially with a “gang” of words that overlap with the target onset but have greater mutual overlap within the gang (e.g., *clock*, *clod*, *clot* all overlap with *clue* in the first two phonemes, but with each other in the first three; see schematic in upper panel of **Figure 8**). When the first phonemes of the target (e.g., /kl/) are presented, the gang members are also activated. The target and the gang all benefit from lexical feedback; when /kl/ has been presented, those phonemes get a boost from feedback from the target and the gang. However, the gang members also boost each other by sending feedback to their mutual vowel (/⊃/, substituted with /a/ in TRACE), even though it has not been presented. This gives these items an activation advantage over the target until enough of the vowel in the input has been presented to allow the target to overcome inhibition from the gang. It may help to visualize this set of items in a tree structure, where *clue* and the /kl⊃/ gang are on the same branch through the second consonant and then branch apart at the vowel (lower panel of **Figure 8**). Since any of these nodes in TRACE can inhibit each other, the gang effect benefiting the *clock–clot–clod* branch allows the gang members to inhibit items on the sparser branch where *clue* is located, temporarily overwhelming benefits of feedback to the *clue* branch and slowing recognition relative to the no-feedback condition.

Strauss (2006) found that whether negative ganging leads to a feedback disadvantage depends further on the size and composition of the target’s competitor neighborhood (in particular, its cohort), the target’s length, and the amount of overlap with the gang members. Gang effects are more likely to be negative when the target is short, the cohort is large, and there is a high degree of overlap among the gang members. However, we say “more likely” because the direction and magnitude of gang effects depends on the other factors we mentioned above, as well as the particular phonemes in the target and potential gangs (see Strauss, 2006, for details). The small phoneme inventory of TRACE leads to relatively dense packing of phonological space, and the relative specificity of the different phonemes varies. The degree of ganging depends on all the factors we have discussed (and possibly additional ones). More generally, this negative ganging effect is a type of phonotactic frequency effect – one that flexibly reflects interactions between the structures of the phoneme and lexical level inventories (rather than rigid specifications of n-phone frequencies, i.e., diphones, triphones, etc.). In other words, interactive activation provides a simple mechanism that captures different orders of prior probabilities at multiple scales.

## General Discussion

Bayes’ theorem provides a simple formalism for optimal integration of current input and prior knowledge for perceptual and cognitive inference under uncertainty. Conceptually, the theorem specifies a straightforward way to optimally estimate the probability of an outcome given some evidence (that is, its conditional probability, or the probability of the outcome given the evidence) based upon the probability of the evidence given the outcome and the independent probabilities of the outcome and evidence (the probability of the outcome, whether or not the evidence is observed, and the probability of the evidence, whether or not the outcome is observed). Numerous studies have shown that cognitive systems approximate Bayesian inference (Clayards et al., 2008; Feldman et al., 2009; Chater et al., 2010; Sonderegger and Yu, 2010; Kleinschmidt and Jaeger, 2015). However, it is unlikely that the human brain literally calculates Bayes’ theorem rather than approximating it in cases where optimal or near-optimal performance is observed (Oaksford and Chater, 2007; Geisler and Ringach, 2009; Sanborn et al., 2010; McClelland et al., 2014), so what are the cognitive (and neural) mechanisms that produce Bayesian or Bayes-approximate behavior? One candidate is interactive activation: bi-directional information flow allows cognitive systems to flexibly learn to approximate optimal integration of bottom-up input with top-down prior knowledge (McClelland et al., 2014). One specific prediction from this view is that top-down feedback should improve performance, especially in noisy conditions. This prediction was challenged by a set of simulations (FP98) that found no benefit of feedback – a result that has played a key role in arguments that interactive activation is not a viable implementation of Bayesian inference in spoken word recognition (Norris et al., 2000, 2016). In this paper, we re-evaluated this prediction using a systematic analysis of spoken word recognition in the TRACE model (McClelland and Elman, 1986) with and without feedback, and at varying levels of noise.

When we examined the performance of TRACE on a large number of items under clear and noisy conditions, feedback clearly facilitated word recognition. Overall, feedback increased speed and accuracy of word recognition for both clear and noisy inputs. This invalidates the argument Norris et al. (2000) made against interactive activation based on FP98 and supports interactive activation as a viable cognitive (and perhaps neural) mechanism for implementing Bayesian inference.

One might argue that TRACE with feedback turned off is not a fair implementation of an autonomous model. In point of fact, such a model would actually represent the largest autonomous network model that has been implemented. The Merge simulations Norris et al. (2000) report were conducted with only six input phoneme nodes, four phoneme decision nodes, and four word nodes, and no representation of sequential order (so it would not be able to distinguish the words “cat,” “act,” and “tack”). Thus, TRACE without feedback represents an autonomous model able to operate with a relatively large lexicon and phoneme inventory, while representing temporal order. The model would not predict lexical effects on phoneme nodes. Lexical effects on phoneme decisions are modeled in Merge with special-purpose phoneme decision nodes. However, because those phoneme decision nodes do not interact with phoneme or word nodes, they are not relevant for modeling word recognition, which was the aim of our simulations.

With clear evidence in hand for a helpful role of feedback in interactive models, we turn next to several theoretical issues raised by Norris et al. (2000, 2016). These issues are critical because they form the core of the argument, advanced by Norris et al. (2000), that feedback is not a viable cognitive algorithm or, at best, feedback provides an inferior implementation of Bayes’ theorem. We will address the relative parsimony of interactive vs. autonomous models as framed by Norris et al. (2000), whether feedback would hinder perceptual processing (and related misconceptions of models like TRACE), growing neural evidence supporting feedback, and varieties of feedback that Norris et al. (2016) claim are acceptable and fundamentally distinct from “activation feedback” in TRACE.

### Occam’s Razor and the Feedback Debate

**FIGURE 9:**
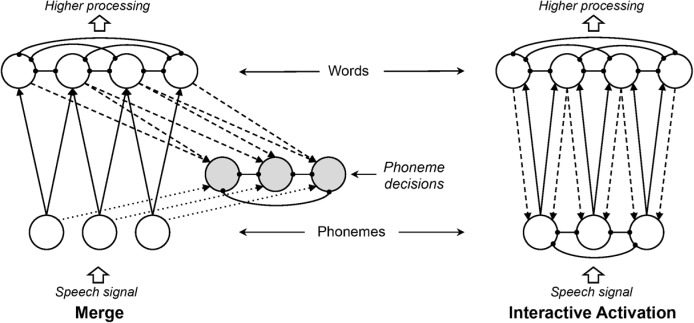
Elaborated schematics of Merge and an equivalently simplified interactive activation model (IAM) for purposes of comparing model complexity in terms of numbers of nodes and connections. Arrows and knobs indicate excitatory and inhibitory connections, respectively. Dashed lines indicate excitatory connections that provide lexical feedback in the IAM but feed forward (or “sideways”) to phoneme decision nodes in Merge. Gray nodes indicate additional nodes required by Merge, and dotted lines indicate additional connections required by Merge. As discussed in the text, feedback connections in the IAM provide emergent prelexical sensitivity to phonotactics; such sensitivity in Merge would require a tremendous proliferation of connections. Thus, the obviously more compact IAM model provides all functionality the larger Merge model does while providing phonotactic sensitivity as an emergent property. Note that neither of these networks would be valid models of spoken word recognition, as they cannot encode temporal order (e.g., CAT, ACT, and TACK would all be equally activated by the input strings /kæt/, /ækt/, or /tæk/, as well as by /ætk/, /ktæ/ or /tkæ/). This remains a problem for the Merge model. Differences in complexity may be even greater in systems that address this dilemma.

Norris et al. (2000) motivate their arguments against feedback on parsimony: “The principle of Occam’s razor instructs theorists never to multiply entities unnecessarily” (p. 299). Another way of framing Occam’s razor is to prefer the simpler of two explanations that account for the same data. How do we determine simplicity? In the case of neural network models, intuitive bases for comparison are the numbers of nodes and connections. Let’s begin with Merge and the sort of simplified IAM Norris et al. (2000) compared. The elements of the models are nodes and connections. **Figure 9** presents an elaborated version of **Figure 1**, depicting nodes and connections in simple versions of Merge and an IAM with three phonemes and four words.

The first thing to note is that Merge requires more nodes and connections than an equivalently simplified IAM; in **Figure 9**, these added nodes are shaded gray, and the added connections are depicted with dotted lines. Let *P* be the number of phonemes, *W* the number of words, *L* mean word length (mean phonemes per word). Merge has 2*P* + *W* nodes; the IAM has *P* + *W* nodes. The extra *P* nodes in Merge are the phoneme decision nodes.^4^ Both models have *P*! / [2(*P* - 2)!] + *L*! / [2(*L* - 2)!] inhibitory connections. The IAM has 2*WL* excitatory connections (one feedforward and one feedback connection for every phoneme-to-word link). Merge has this number of connections but an additional *P* connections (from phoneme input nodes to phoneme decision nodes; see also the caveat in footnote 3). Thus, just based on a basic complexity comparison, the models are not equivalently simple; the IAM requires fewer nodes and fewer connections.

Dashed lines in **Figure 9** indicate connections that afford feedback in the IAM but feed “sideways” to phoneme decision nodes in Merge. We say “sideways” because the phoneme decision nodes are a set of terminals; the layer of decision nodes has no purpose except to integrate bottom-up and top-down information without allowing “feedback activation.” Norris et al. (2000) note that phoneme decision nodes allow Merge to be a “multiple outlet” system (Cutler et al., 1987) and have suggested that it is a problem that models like TRACE do not have explicit nodes for phoneme decisions. As such, they present a strong theoretical commitment that phoneme-level and lexical-level information is not merged for any functional purpose except for metalinguistic decisions about phonemes. By contrast, in TRACE (as in virtually all connectionist models), it is assumed that attention can be directed to phoneme or lexical levels when decisions are required about one or the other; the Luce (1959) choice rule is typically applied to activations to make such decisions. If, as Norris et al. (2000) posit, nodes cannot serve the dual purpose of processing and decisions (i.e., if nodes cannot be read-out unless they are designated as decision nodes), any functional level one wishes to link to explicit behavior will require appending a layer of decision nodes (i.e., Merge requires a layer of word decision nodes, as would any other model). But note that there is still no mechanism built into decision nodes that “reads” activations in order to execute a decision; a choice process (e.g., applying the Luce choice rule, or applying a simple winner-take-all rule of choosing the decision corresponding to the most active node) still must be added. Thus, this is not a point of true difference in models.

Furthermore, there are also aspects of Merge that are not included in **Figure 9** (and so far as we are aware, have never been implemented). Phonotactic sensitivity emerges in an IAM like TRACE thanks to lexical feedback; the more words there are that contain a particular multi-phoneme pattern, the more feedback resonance will accumulate for that pattern. (For neural evidence that phonotactic sensitivity arises from lexical feedback, see Gow and Olson, 2015.) Norris et al. (2000) have appealed to the possibility of building in various kinds of prior phonotactic knowledge in the forward connections from phonemes to words or via lateral connections within the phoneme input layer. This would allow Merge to have sensitivity to phonotactics at multiple scales without feedback. For example, to account for lexically mediated compensation-for-coarticulation, McQueen (2003) speculated that transitional probabilities of different orders (diphones, triphones, etc.) could be wired into the phoneme layer of a model like Merge. Although the mechanism for adding transitional probability connections to Merge has never been specified, even adding first-order (diphone) connections would require *P*^2^-*P* connections (1,560 connections for 40 phonemes). Adding triphones would require *P*^3^–*P*^2^ connections (62,400 connections for 40 phonemes). Quadraphones (a possibility not excluded by McQueen’s speculations) would require 2,496,000 connections for 40 phonemes. Higher-order *n*-phones or more complex possibilities (e.g., diphones conditioned on *n*-phones) would obviously expand the number of connections even further.

Given the increase in model elements required by autonomous architectures, how is Merge simpler? Norris et al. (2000) assert that feedback is inherently illogical and complex. For example, Norris et al. (2000) say:

The logic of the process requires information to flow in one direction: from sounds to words. This direction of information flow is unavoidable and necessary for a speech recognition model to function (p. 299).

And Norris et al. (2016) assert:

Feedback performing Bayesian computations may be considered unobjectionable; however, it is incontrovertibly simpler to perform those same computations without feedback (p. 5).

We reject the assertions that feedback is illogical or that feedback “incontrovertibly” imposes unspecified complexity (that is, complexity that cannot be quantified by, e.g., enumerating nodes and connections). Norris et al. (2016) do not present or cite mathematical principles to support these assertions and deny the logical implications of the abundance of feedback connections in neural systems (see section “Neural Data Relevant to the Feedback Debate” below). Without a case that interaction is more complex based on model size or computational principles, their claims rest completely on the argument that feedback cannot benefit perception and that feedback in fact will hinder perception. Given our demonstrations of a helpful role of feedback, we turn next to the question of whether feedback in a model like TRACE hinders perception.

### Does Feedback Hinder Perception?

Norris (1994) and Norris et al. (2000, 2016) have repeatedly claimed that feedback in TRACE leads the model to “hallucinate.” Here is a representative passage from Norris et al. (2000).

If the /n/ in the middle of *phoneme* cannot be distinguished clearly by the phoneme level alone, interactive bias from the lexical level can boost the activation for the /n/ and make it more likely that the phoneme will be identified. Of course, if the input were the non-word *phomeme* instead, the biasing effect would impair performance rather than help it, in that the mispronunciation would be overlooked. That is, interactive bias models run the risk of hallucinating. Particularly when the input is degraded, the information in the speech input will tend to be discarded and phonemic decisions may then be based mainly on lexical knowledge (p. 302).

As we have reviewed elsewhere (Magnuson et al., 2012, 2013), Figures 6, 7, 13 and 30 of the original TRACE paper (McClelland and Elman, 1986) show that TRACE balances top-down and bottom-up information such that the model exhibits bottom-up priority. A key example in the original paper was based on *plug-blug-?lug* (that is, a word [plug], a similar non-word beginning with /b/, and a stimulus with the first segment ambiguous between /p/ and /b/). We can extrapolate to the example of “phomeme:” The word “phoneme” would be highly activated given this input, but less so than when given “phoneme.” Input will *not* “tend to be discarded” nor will “the mispronunciation … be overlooked,” as activations at the phoneme level will clearly reflect the competing influences of bottom-up and top-down activations (/n/ will become much more active than it would in a case where it was not favored by a lexical entry, e.g., numb/none, and while /m/ would be strongly activated, and in particular, much more so than it would be given a clear input of “phoneme,” it would be less activated than it would be by a clear bottom-up input corresponding to /m/). Mirman et al. (2005) examined this specific issue closely. They found that feedback in TRACE could not overwhelm bottom-up input but could delay recognition of lexically inconsistent phonemes when there is some bottom-up ambiguity, as in the ‘phomeme’ case, where the similarity between lexically consistent /n/ and acoustically present /m/ creates sufficient bottom-up ambiguity for the top-down feedback to delay recognition of the /m/. Experiments with human subjects confirmed lexically induced delays in phoneme recognition under the conditions predicted by TRACE.

In the quote above, another misconception about interactive activation is highlighted in the notion that the effect of feedback will only be to boost the activation of /n/ given “phomeme” as input. Norris et al. (2016) similarly claim that “in TRACE, activation is fed back via top-down connections to simply boost the activation of nodes at an earlier level” (pp. 9–10). However, due to lateral inhibition and subsequent inter-layer resonance, boosting one phoneme has myriad, complex effects. The idea that feedback simply boosts activations of lexically consistent phonemes oversimplifies the actual dynamics of an IAM.

The hallucination claim also implicitly underestimates the propensity for misperceptions in human listeners. In fact, there is a close concordance between TRACE’s tendency to “hallucinate” lexically consistent phonemes and the tendencies in misperceptions by human listeners in a study of lexically induced phoneme inhibition (Mirman et al., 2005). This finding is consistent with other examples of context-induced misperceptions, such as to failures to detect mispronunciations (Cole, 1973; Marslen-Wilson and Welsh, 1978), phoneme restoration (Samuel, 1981, 1996, 1997), and related findings in other domains, such as illusory contours in visual perception (Lee and Nguyen, 2001). It is crucial to recognize the distinction between *optimal* performance (the best possible under particular conditions) and *perfect* performance. The benefit of feedback is to approximate optimal use of context-appropriate prior knowledge. The present simulations showed that feedback can indeed slow word recognition, but this happens under particular conditions (embedded words, negative gang effects, etc.) when prior knowledge conflicts with bottom-up input. As in the case of lexically induced delays in phoneme recognition (Mirman et al., 2005), these cases of feedback hindering perception are not valid arguments against feedback because they are consistent with Bayesian integration of bottom-up input and prior probability: When the input conflicts with prior knowledge, perception *should* be more difficult. McClelland et al. (2014; also Movellan and McClelland, 2001) demonstrated that it is possible to balance feedforward and feedback gain to avoid hallucination and produce optimal (Bayesian) performance. The tradeoff between potential misperceptions and positive benefits of feedback must be balanced through proper weighting of bottom-up and top-down information sources – as it is in TRACE.

### Neural Data Relevant to the Feedback Debate

Norris et al. (2016) have argued that in general, neural-level data does not bear heavily on the feedback question because

… in a theory stated at what Marr (1982) would term ‘computational’ or ‘algorithmic’ levels of analysis, there might be no need for feedback between different stages of processing, even though the requirement to implement the processing computations in neural tissue might best be served by exploiting recurrent connections between layers of neurons … an algorithmic account with no feedback would not need to be altered in the light of evidence of the existence of recurrent neural connections, so long as those neurons were just part of the implementation of that algorithm (p. 4).

Certainly, the presence of recurrent connections in the brain may be compatible with multiple computational accounts, including those that do not include feedback. However, a number of recent studies have provided compelling neuroscientific evidence that may be able to inform the feedback debate at the computational and algorithmic levels of analysis. Myers and Blumstein (2008), for example, examined brain activity related to the classic Ganong (1980) effect. In this effect, phoneme categorization of a word-initial ambiguous token (e.g., between /g/ and /k/) is modulated by the lexical context (e.g., in *–iss* versus *–ift*). In a functional MRI experiment, Myers and Blumstein showed that the Ganong effect modulated neural activity in the bilateral superior temporal gyri (STG), as well as in the left middle frontal gyrus and in the left cingulate. The STG have long been associated with low-level acoustic processing (for a review, see Hickok and Poeppel, 2007), whereas frontal and medial structures are often implicated in decision-making processes (Krawczyk, 2002). Myers and Blumstein argued that the specific pattern of STG activation in their data reflected integration of lexical and acoustic-phonetic information. They further noted that under a strictly feedforward architecture, lexical context would not influence activation in early auditory areas. Norris et al. (2016) have argued that this conclusion in favor of interactive models presupposes that the STG is involved only in prelexical processing and point to fMRI evidence ascribing additional roles (e.g., in lexical processing) for the STG. But indeed, Myers and Blumstein argue that the most likely situation is that the STG is involved in both lexical and prelexical processing; what makes their data inconsistent with autonomous architectures is the fact that STG activation is not related to decision difficulty (e.g., Binder et al., 2004; Blumstein et al., 2005; Myers et al., 2009). Similar effects of semantically biased sentence contexts on phoneme perception (Borsky et al., 1998) have been tied to the left STG (Davis et al., 2011; Guediche et al., 2013), consistent with interactive accounts of speech perception.

Still, lexical processing occurs on a millisecond timescale, and the temporal resolution of fMRI may be inadequate to examine whether activation at the lexical level changes activation at the phoneme level (Hernandez et al., 2002; Spivey, 2016). Results from techniques that allow for both fine-grained spatial and temporal resolution provide even stronger evidence of feedback (Gow et al., 2008; Travis et al., 2013; Gow and Olson, 2016; Leonard et al., 2016). Leonard et al. (2016), for instance, conducted an electrocorticography (ECoG) study in which they directly recorded cortical responses to unambiguous speech (e.g., *faster* [fæstr], *factor* [fæktr]) and to speech in which the disambiguating token was replaced with noise (e.g., [fæ#tr]). Participants tended to perceive the ambiguous stimuli in a bi-stable fashion, sometimes reporting having heard one word and sometimes having heard the other. Critically, when the participant perceived the ambiguous stimulus as one word (e.g., *faster*), the cortical response in the STG at ∼120 ms post-stimulus onset matched the STG response to a clear production of that word at that same time. That is, the online response in the STG to the same acoustic input differed depending on how that instance of the stimulus was perceived. Leonard et al. (2016) also created filters that mapped from cortical patterns of activity to spectrograms; these filters were created based on passive listening to clear productions of speech and then fit to the ECoG data from the main experiment. The spectrograms derived from the ECoG data in the ambiguous condition closely matched the spectrogram for the clear production of the item that the participant perceived. Finally, Leonard et al. (2016) reported that the pattern of cortical activity *prior* to the presentation of the ambiguous stimulus predicted a subject’s perception on that trial and that this “pre-stimulus neural bias” (i.e., set of prior expectations) was heavily associated with the left inferior frontal gyrus, an area associated with higher level aspects of cognition. Strictly feedforward models would posit neither the pattern of activation in the STG nor that prior expectations would restore intact perception of a stimulus.

Taken together, these findings offer compelling neural evidence that is readily explainable through interactive architectures but not apparently compatible with purely feedforward accounts. On our view, these sorts of neural data should inform theories at other levels of processing in Marr’s (1982) hierarchy. Consider again the claim by Norris et al. (2016) that neural evidence for activation feedback would not require alteration of an algorithmic (or presumably, computational) level theory that rejects feedback.

Encapsulation of computational and/or algorithmic theory is problematic for several reasons (Cooper and Peebles, 2015). For example, if neural evidence is irrelevant to computational-level accounts, behavioral evidence may also be irrelevant (Griffiths et al., 2008). After all, human behavior is subject to all sorts of performance factors that might just be side-effects of one of many possible algorithms or implementations (this will be familiar to readers acquainted with the competence-performance distinction in linguistics, e.g., Chomsky, 1965 vs. Seidenberg, 1997). More importantly, Marr’s (1982) proposal was that a complete explanation requires an account at all three levels of analysis – computational, algorithmic, and implementational. Thirty-five years later, after substantial progress at each of the levels, we have opportunities to develop theories that bridge levels of analysis: theories that specify the computation, the algorithm for making that computation, and the neural implementation of that algorithm. We agree with Oaksford and Chater (2007) that even comprehensive rational theoretical accounts will need to be constrained by evidence for the details of algorithms and neural implementation. Each level should constrain the others as we approach comprehensive and biologically plausible explanations, resulting in a multi-level theory rather than independent theories at each level. Interactive activation is one such multi-level theory: At the computational level it is Bayesian inference (or Bayes approximate, depending on details); at the algorithmic level, mappings are achieved through complex interactions of simple elements with forward, backward, and lateral connections; and it relates at the implementational level directly to neural feedback.

### Good Online Feedback, Bad Online Feedback

At issue in the interactivity debate is whether there is feedback from higher levels of processing that influences the activation levels of lower layers during online processing. As such, Norris et al. (2016) have argued that there are other types of feedback that are compatible with their perspective, including feedback for learning, feedback for attentional control, feedback for “binding” (based on an argument that it is necessary for lower levels of representation to explicitly encode parsing that emerges at higher levels^5^), generative models, and predictive coding (Rao and Ballard, 1999).^6^ More generally, they say that “feedback performing Bayesian computations may be considered unobjectionable” (p. 10) but add that “it is incontrovertibly simpler to perform those same computations without feedback” – a considerable shift from the strong position that feedback cannot possibly help.

Norris et al. (2016) further cite examples of systems that operate without feedback, such as deep learning networks for speech recognition (Hinton et al., 2012), as if to suggest that if such systems operate without online feedback, then feedback is unnecessary. When they cite Hinton et al. (2012), they say “the best current automatic speech recognition systems are feed-forward” (p. 5). They do not mention that in the system described by Hinton et al. (2012), as in many deep learning systems, there are phases of training that employ feedback, and not just “feedback for learning” (that is, backpropagation of error); these systems use what Norris et al. (2016) call “activation feedback” during training. Norris et al. (2016) later acknowledge this regarding a related system (Hinton et al., 2006) and conclude that “feedback may sometimes be necessary to learn a code but, once established, that code can be used without feedback” (p. 12; note that the feedback at issue here is again “activation feedback”).

Indeed, learning is a key concern. Friston (2003) argues that for any system that is “not easily invertible” (one where there is a many-to-many mapping from inputs to outputs, as in spoken language), a feedforward solution is possible but will be nearly impossible to discover without supervised learning or feedback. Friston (2003) describes “empirical Bayes,” a variant of Bayesian learning in which higher levels in hierarchically organized representations provide provisional prior probabilities for lower levels via feedback – even when the higher-level representations are imprecise early in learning (see Magnuson, 2008, for a more detailed discussion of Friston’s arguments and their implications for the interaction debate, and Grossberg, 2003, for related arguments).

One way to construe feedback in interactive-activation models is that it makes the models implicit Bayesians. For example, McClelland (1991) and Movellan and McClelland (2001) show how stochastic interactive models provide optimal integration of context and bottom-up stimulation (see also Grossberg, 2003, for strong arguments regarding the need for resonance/interaction/feedback to account for perception in multiple domains, and a review of neurophysiological findings demonstrating top-down effects and connectivity at multiple levels of neural systems). The addition of feedback gives the model early and continuous access to dynamic, context-sensitive prior probabilities at multiple windows of analysis without explicit representations of the probabilities. For example, as discussed above, transitional probabilities will emerge as a function of the structure of lexical neighborhoods: The more words that contain a particular sequence, the more feedback the component phonemes of that sequence receive. In the case of weak bottom-up information (e.g., due to a low amplitude input signal or the presence of noise), feedback will help. Given roughly equivalent evidence for two sublexical alternatives, if one is contained in a word and the other is not, or one is contained in more words than the other, feedback will push the system toward the more frequent (likely) alternative. Given roughly equivalent bottom-up information for two lexical alternatives, if one has a higher prior probability (either in terms of lexical frequency [Dahan et al., 2001a, for implementations of frequency in TRACE] or sublexical frequencies implicit in the lexicon), this will be reflected in greater feedback and will push the system to favor the alternative more consistent with prior knowledge.

McClelland (2013) and McClelland et al. (2014) have demonstrated how an IAM can be made fully Bayes-consistent by allowing sublexical nodes to keep track of their contribution to lexical activation and to remove that contribution from the feedback they receive.^7^ Norris et al. (2016) claim that adding such a mechanism “adds an assumption to the core set of interactive-activation assumptions” and therefore “Interactive activation, with its activation feedback, can thus be viewed as the wrong place to start in model development. All we actually need is Bayes” (p. 9). But that is not quite all, because on their account, we also need feedback for learning, plus feedback for binding, plus feedback for attentional control, plus feedback for predictive coding. Adding a mechanism to remove a node’s contribution to feedback it receives, as Khaitan and McClelland (2010), McClelland (2013), and McClelland et al. (2014) have proposed, is principled (consistent, e.g., with constraints on unidirectional causal graphs [Pearl, 1982; see McClelland, 2013, for discussion]). It is not a radical reconceiving of interaction. It is an example of standard scientific practice in model development (and again, it is not the only way to make a model like TRACE Bayes-equivalent).

While we think the designation of “activation feedback” as a separate category of feedback distinct from the others in the Norris et al. (2016) list of “unobjectionable feedback” is in error, if we return to the question of parsimony, it seems that the arguments in favor of online feedback continue to accrue. Recall that feedback in TRACE provides emergent phonotactic sensitivity, which would have to be added in an as-yet unspecified manner in Merge or either Shortlist model, possibly requiring a rather dramatic increase in connections (as we discussed above), or other elements that would increase complexity in the case of a computational-level Bayesian model like Shortlist B (Norris and McQueen, 2008). Furthermore, many of the “variants” of feedback that Norris et al. (2016) find unobjectionable would be accommodated by the type of feedback implemented in a typical IAM like TRACE. Lexical feedback provides a natural pathway for attentional modulation. Lexical feedback actually provides a rough approximation to the “feedback for binding,” Norris et al. (2016) suggest is needed (though we disagree with the need for the *explicit* and complete top-down “binding,” Norris et al. (2016) describe as necessary; see footnote 5); in TRACE, the words most activated at the lexical level will continuously push phoneme activations toward the sequence of phonemes most consistent with the emergent parse at the lexical level.

But finally, consider the formal contribution of feedback in a model like TRACE. As McClelland (2013) discusses in detail, feedback provides a generative model. A word node sending feedback to its constituent phonemes is a case of a higher-level element sending top-down feedback to its “inferred” (embodied) causes (the specific phoneme nodes that would activate the word node). Even the original, deterministic implementation of TRACE will come close to approximating an optimal Bayesian analyzer (Movellan and McClelland, 2001; McClelland, 2013); lexical representations encode sublexical patterns of varying grain sizes that guide the system as a whole toward the most likely cause of a particular input pattern. Given noisy input that is consistent with two sublexical patterns, one of which occurs in one or more lexical items but the other of which does not occur (e.g., a segment midway between /s/ and /∫/ preceding /tr/, where /str/ is a likely sequence in English but /∫tr/ is not), lexical feedback provides a generative model that guides the system to a rational response given the input and top-down knowledge about likely patterns embodied in the lexicon.

### Quod Feedback?

Our simulations demonstrate how feedback can help recognition (see also McClelland et al., 2006 and the resulting debate between McQueen et al., 2006, and Mirman et al., 2006; and McClelland et al., 2014). Norris et al. (2016) argue that this debate has been effectively resolved by the development of Bayesian models of perception and cognition. However, short of committing to a cognitive (and neural) mechanism that literally calculates Bayesian posterior probabilities, the question remains: how are such computations implemented in the minds and brains of actual humans? While Norris et al. (2016) claim that humans are literally ideal observers (that is, we perform optimal perception), the conventional ideal observer approach (e.g., Geissler, 2003) is to assess what an optimal system would achieve for a given stimulus and to compare biological performance to the ideal – with the assumptions that a biological system may approximate the ideal and that differences between biological performance and the ideal may provide insights into the mechanisms supporting biological perception (which may or may not be Bayes-approximate or Bayes-equivalent) and other relevant details (such as degree of internal noise). We believe that feedback is a strong candidate for implementing Bayesian computations at multiple levels of analysis.

First, let us be clear about the term “feedback,” given that Norris et al. (2016) have suggested there are at least six types of feedback.

(1) Activation feedback
(2) Attentional modulation
(3) Feedback for learning
(4) Feedback for binding
(5) Feedback in service of generative models
(6) Error signals fed back in predictive coding

Of these, they find objectionable only “activation feedback”: the ability for one level of a hierarchical network to alter the states of inferior levels during processing. As formulated by Norris et al. (2000, 2016), the arguments against feedback were:

(1) Feedback from level *L* to an inferior level cannot improve performance at level *L* compared to a system without feedback.
(2) Not only can feedback not help, it hinders perception by causing hallucination.
(3) A system without feedback is more parsimonious than a model with feedback because (a) logically, information should only feed forward (Norris et al., 2000, p. 299) and (b) “it is incontrovertibly simpler to perform [the] same computations without feedback” (Norris et al., 2016, p. 5).

We have responded to all three of these claims. To summarize:

(1) *Feedback improves performance*. Our simulations demonstrate large, robust advantages for feedback, especially when noise is added to inputs for TRACE.
(2) *Feedback does not entail “hallucination.”* Both formal analysis (McClelland et al., 2014) and empirical testing (Mirman et al., 2005) demonstrate that interactive activation models in general, and the TRACE model in particular, appropriately balance bottom-up and top-down information. Such models do not “discard” bottom-up input in favor of feedback-consistent perception (see our above of examples from the original TRACE paper, e.g., Figures 6, 7, 13, and 30 from McClelland and Elman, 1986); however, prior knowledge can hinder recognition of input that violates expectations – *consistent with Bayesian inference*.
(3) *A system with feedback is more parsimonious than a model without feedback*. As we detailed, based on simple enumeration of required nodes and connections, an interactive model is simpler than an autonomous analog. An interactive model would also account for substantially more phenomena, because lexical feedback provides emergent sensitivity to phonotactics, and other forms of prior probability that would have to be somehow added in feedforward connections in models like Merge (Norris et al., 2000). Considering the six types of feedback, connections providing lexical feedback could do at least quadruple duty, providing the pathway for attentional modulation, binding, and already embodying a generative model (cf. McClelland, 2013), in addition to providing activation feedback. All of these mechanisms would have to be somehow added to autonomous models. Parsimony clearly favors feedback.

The autonomy position has shifted from “feedback is never necessary” and “feedback cannot improve processing” to, essentially, feedback of any sort *except* activation feedback can be helpful (“These forms of feedback [attentional, learning, binding] either are necessary for perception to be successful, or can make perception more efficient,” Norris et al., 2016, p. 8). Norris et al. (2016) even allow that activation feedback may be helpful for learning (Friston, 2003; Serre et al., 2007); though they still maintain that it should be turned off at some unspecified point. Our simulations show that feedback promotes robust performance in the face of noise – an important piece of the broader mosaic showing that feedback provides a parsimonious mechanism for implementation of Bayesian inference.

## Author Contributions

JM conceived the project, conducted simulations, analyzed results, and led manuscript writing. DM conducted simulations, analyzed results, and collaborated on manuscript writing. SL collaborated on manuscript writing. TS conducted simulations, developed special purpose computer code, and collaborated on manuscript writing. HH collaborated on project development and manuscript writing.

## Conflict of Interest Statement

The authors declare that the research was conducted in the absence of any commercial or financial relationships that could be construed as a potential conflict of interest.

## Footnotes

1 https://magnuson.psy.uconn.edu/jtrace/

2 Without noise, most words in the lexicon were recognized well before cycle 95. Thus, 100 cycles provided adequate time for recognition; accuracy would not significantly increase at any feedback-x-noise level were we to run the simulations for more cycles. The items that were not correctly recognized typically fell short of the threshold and could never reach it (i.e., extending the simulation for more processing cycles would not allow the target to reach the threshold).

3 It is unclear what statistical tests would be appropriate for these data, given that we have conducted simulations with every item in each lexicon. However, the trends are clear and large.

4 We are assuming a static layer of decision nodes for mathematical convenience. Norris et al. (2000) indicate that decision nodes would instead by wired-up as needed. The machinery for doing this on-the-fly wiring would require an unspecified amount of additional elements, making the difference in model elements (nodes and connections) even greater.

5 Norris et al. (2016) give the example of phonemic input /habbbdIdddr/, which can be parsed as “hi dear,” “hide ear,” or “hide deer.” They claim that a sublexical level of representation must be informed of the parse that results (“the word *dear*… does not ‘know’ which phonemes activated it … If there are no backward connections, then there is no way of forming an integrated percept of the word *dear* and its constituent phonemes. In order to form such a percept the phonemes need to be bound to the word, and this requires some form of bi-directional communication” (p. 8). We find this suggestion to be at odds with the vast majority of computational cognitive and neuroscience, where there is no need for such discrete identification at lower levels of encoding. In a model like TRACE or Shortlist, without any such “feedback for binding,” the system will settle on a lexical parse based on, e.g., word frequency and (in the case of TRACE) probabilistic phonotactics that emerge from lexical-phonemic feedback.

6 Norris et al. (2016) have claimed that Simple Recurrent Networks (SRNs; Elman, 1991) do not include online, interactive feedback. However, the input to the hidden units at any time step is the current bottom-up input and the results of hidden unit processing of bottom-up and context information from the previous time step transformed by trained context-to-hidden weights. One could argue that this does not qualify as feedback, as it is not literally feedback from explicit lexical units. However, if hidden units influence their own inputs, then there is no sense in which the bottom-up input to the hidden units is encapsulated from the top-down input from the context units; the hidden unit activations cannot be unmixed to identify which portion of the input came from bottom-up inputs or context units (without knowing both the values of connection weights and the activations of the inputs and context units). To claim that the hidden unit states do not constitute feedback because they are only a partial result of the process that yields lexical activations does not accurately represent the dynamics of SRNs.

7 Note that McClelland (1991) and Movellan and McClelland (2001) have shown how variants of interactive activation models with continuous activation (that are made non-deterministic by adding noise to model inputs or internal layers) – and without a mechanism for nodes to deduct their self-contributions to their own feedback – can approximate (or under certain conditions are equivalent to) Bayesian inference (as demonstrated by their ability to exhibit logistic additivity).
